# Population Growth of the Cladoceran, *Daphnia magna*: A Quantitative Analysis of the Effects of Different Algal Food

**DOI:** 10.1371/journal.pone.0095591

**Published:** 2014-04-21

**Authors:** Jong-Yun Choi, Seong-Ki Kim, Kwang-Hyeon Chang, Myoung-Chul Kim, Geung-Hwan La, Gea-Jae Joo, Kwang-Seuk Jeong

**Affiliations:** 1 Department of Biological Sciences, Pusan National University, Busan, Republic of Korea; 2 Departments of Environmental Science and Engineering, Kyung-Hee University, Yongin, Republic of Korea; 3 Institutes of Environmental Ecology, Chemtopia Co. Ltd., Seoul, Republic of Korea; 4 Department of Environmental Education, Suncheon National University, Suncheon, Republic of Korea; 5 Institute of Environmental Science & Technology, Pusan National University, Busan, Republic of Korea; University of Shiga Prefecture, Japan

## Abstract

In this study, we examined the effects of two phytoplankton species, *Chlorella vulgaris* and *Stephanodiscus hantzschii,* on growth of the zooplankton *Daphnia magna*. Our experimental approach utilized stable isotopes to determine the contribution of food algae to offspring characteristics and to the size of adult *D. magna* individuals. When equal amounts of food algae were provided (in terms of carbon content), the size of individuals, adult zooplankton, and their offspring increased significantly following the provision of *S. hantzschii*, but not after the provision of *C. vulgaris* or of a combination of the two species. Offspring size was unaffected when *C. vulgaris* or a mixture of the two algal species was delivered, whereas providing only *S. hantzschii* increased the production of larger-sized offspring. Stable isotope analysis revealed significant assimilation of diatom-derived materials that was important for the growth of *D. magna* populations. Our results confirm the applicability of stable isotope approaches for clarifying the contribution of different food algae and elucidate the importance of food quality for growth of *D. magna* individuals and populations. Furthermore, we expect that stable isotope analysis will help to further precisely examine the contribution of prey to predators or grazers in controlled experiments.

## Introduction

Cladocerans in freshwater ecosystems are among the most important biological entities that contribute to the complexity of food web structure and function [1]. They are typically primary consumers that utilize phytoplankton as their food source. Many species of cladocerans are filter-feeders that obtain food from the water by filtration [2], [3], and are sometimes used as control agents against phytoplankton proliferation in a method known as biomanipulation [4], [5]. Some cladocerans consume organic particles or rotifers [6], [7]; however, most species rely on energy obtained from phytoplankton for population growth.

The nutrient content and biomass of prey phytoplankton are important factors in cladoceran growth [8], [9]. Therefore, the quality and quantity of phytoplankton consumed [10], [11] are crucial factors controlling the growth of cladoceran populations. Previous studies have investigated the size and morphology of algal species (e.g., [12], [13]), and Ahlgren et al. [8] provided comprehensive data on phytoplankton nutritional status. These studies suggest that the quality of prey phytoplankton affects cladoceran population growth. Urabe and Waki [14] provided evidence that changes in biochemical composition of the diet clearly affected the growth of herbivorous species such as *Daphnia*. However, comparisons of algal growth and composition with cladoceran growth are required to quantify the direct contribution of algal intake to cladocerans.

Even if highly nutritious algae are available, algae do not contribute to growth of individuals or populations unless high assimilation rates are maintained, and the majority of this energy resource will be confined to the gut contents and ultimately ejected. Determining the quantitative contribution of prey to consumers is challenging, and few studies addressed this topic [15], [2], [16] prior to the emergence of stable isotope analysis. Phillips and Koch [17] recommended the isotope mixing model, which enables determination of the contribution of the most abundant algal species to growth of an individual consumer. Although the stable isotope signature does not accurately represent assimilation rate, results obtained using this approach can provide information on the quantitative contribution of prey to the consumer, which can be recognized as the assimilation rate. Stable isotope analysis can be used to explain the contribution of algal species to the population growth of cladocerans.

In this study, we experimentally investigated the relationship between a cladoceran species and its prey phytoplankton from the perspective of the algal contribution to offspring characteristics and size of adult individual zooplankton. Two phytoplankton species, *Chlorella vulgaris* and *Stephanodiscus hantzschii*, were used as food algae; the zooplankton studied was the cladoceran *Daphnia magna*. Offspring characteristics and adult individual size were measured. *Daphnia magna* is one of the most popular herbivorous cladocerans for use in culture experiments and *C. vulgaris* is frequently used in *Daphnia* growth experiments. *Stephanodiscus hantzschii* is an important phytoplankton species, particularly in Far-East Asian countries, where the species proliferates in the winter [18], [19], [20]. Therefore, these species were conducive to understanding the contribution of two algal species to a common grazer. Stable isotope analysis was conducted to quantitatively determine the contribution of food algae to *D. magna*.

## Materials and Methods

### Plankton Subculture

*D. magna* obtained from the National Institute of Environmental Research (NIER) of South Korea were grown in Elendt M4 medium [21] in a growth chamber (Eyela FLI-2000, Japan) at 20°C, with a 12L:12D light-dark cycle. Subcultures of the green alga *C. vulgaris* (strain number UMACC 001) and the diatom *S. hantzschii* (strain number CPCC 267) were maintained in a growth chamber (Eyela FLI-301N, Japan) at 10°C, with a 12L:12D light-dark cycle. *S. hantzschii* tolerates a wide range of temperatures, but favors relatively low temperatures [22], [23]. In contrast to *S. hantzschii*, the optimal temperature for *C. vulgaris* growth is >20°C. Excessive population growth often occurs at optimal temperatures, which may affect the constancy of food algae provision (see experimental protocol). Therefore, we maintained the *C. vulgaris* subculture at 10°C for the experiment.

We chose approximately 100 *D. magna* offspring born within 24 h (most ∼0.8 mm long) with similar life-history traits (e.g., birth time and size) from the stock culture, and allowed them to reach the offspring-production stage (hereafter referred as SC, sampled culture). Generally, daphniid females that are larger at birth grow more rapidly and are larger at maturity than those that are smaller at birth [24]. To obtain a similar-sized cohort, we first sorted and eliminated extraordinarily larger or smaller maternal *Daphnia* from the SC (29 individuals were removed). We then randomly selected 30 offspring from the remaining *D. magna* individuals in the SC by adapting a scaled loupe (unit, mm). The 30 adults were between 3 and 4 mm and contained eggs in their brood chamber. We transferred the 30 *D. manga* to a new beaker filled with fresh Elendt M4 medium, and provided sufficient food (*C. vulgaris*) until offspring were produced. In determining the quantity of food algae to provide, we considered the supply level that would be appropriate for zooplankton population growth. Previous research [25] indicated that algal carbon content of approximately 2.5 mg Carbon L^−1^ (units shown as mg C L^−1^ hereafter) in a given volume of zooplankton medium would be sufficient for zooplankton survival and population growth. The first reproduction event occurred at 4 to 5 days after selection. However, the number of neonates from the first reproduction was small, and we used the second clutch from the selected *D. magna* for the experiment. In summary, the initially selected *D. magna* adults were used to produce the offspring employed in the main experimental procedure. The offspring from the second clutch were collected after birth (within 6 h) and were used for the main experiment.

### Experimental Protocol

**Figure 1:**
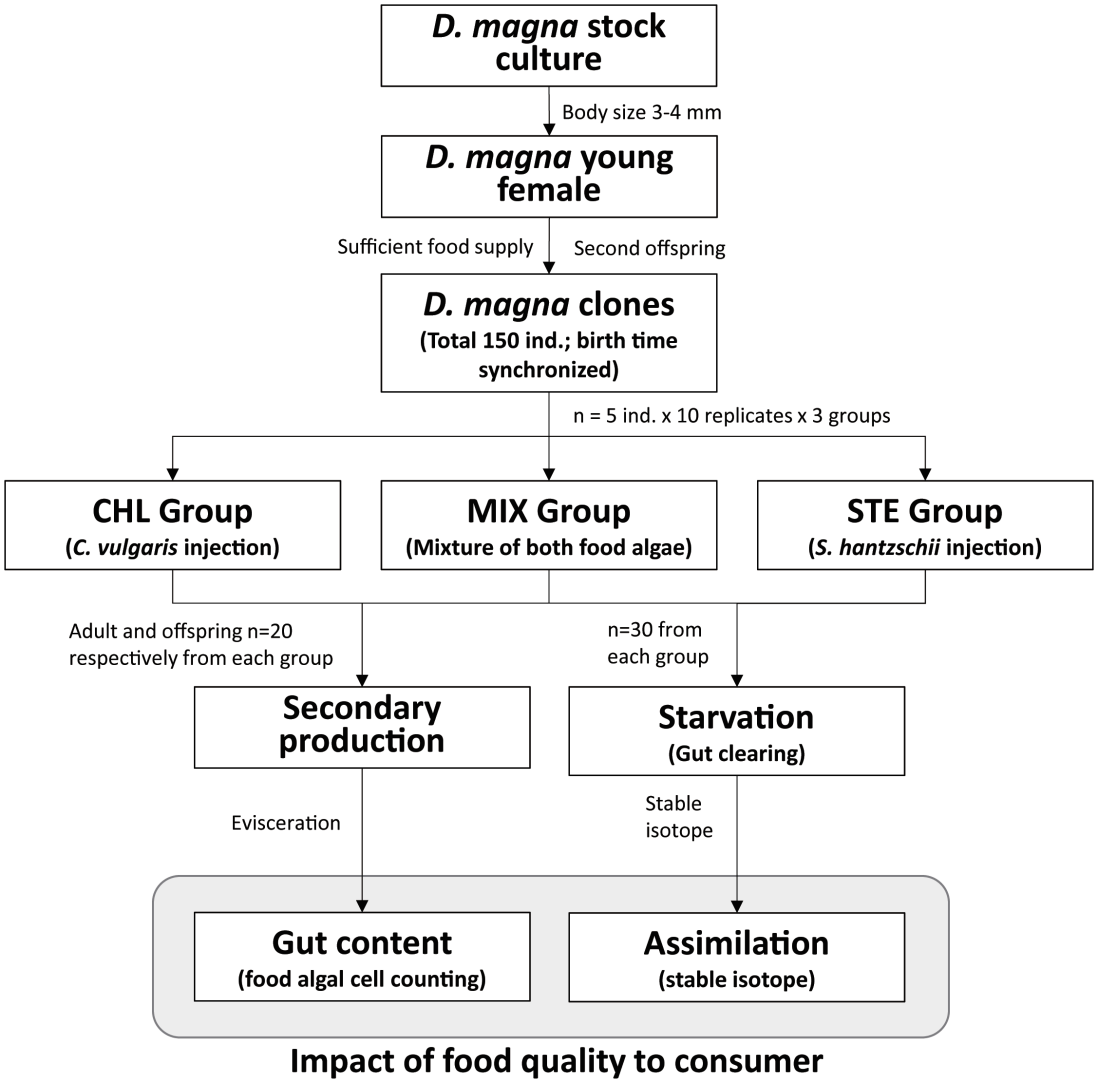
Schematic flowchart of the study. The shaded box indicates analytical processes that used the prepared samples in earlier stages of the experiment.

The overall experimental design is shown in Figure 1. We transferred 150 of the collected offspring into three experimental groups as follows: (1) *C. vulgaris* only (CHL); (2) *S. hantzschii* only (STE); and (3) mixed algae (MIX). For each group, we prepared 10 replicates in 500-mL sterilized beakers filled with 500 mL Elendt M4 medium, and five acclimated *D. magna* individuals were placed in each beaker.

Food algae were supplied at a quantity sufficient to maintain 2.5 mg C L^−1^ in each beaker throughout the experiment. This quantity can be determined from the relationship between algal density and carbon content [25] when algal cell size is known. Thus, before the experiment, we obtained the size information of *C. vulgaris* and *S. hantzschii* by measuring their diameter 50 times, and calculated average size of the algal species (Table 1). Based on the size information, we determined daily food algal supply amount, that the number of cells equal to 2.5 mg C L^−1^ was 8652 cells for *C. vulgaris*, and 8802 cells for *S. hantzschii*. The total daily injection volume was determined accordingly. For example, if daily density of *S. hantzschii* was 687 cells mL^−1^, we injected ca 12.81 mL of *S. hantzschii* stock (687 cells mL^−1^ * 12.81 mL = 8800 cells). The density of food algae changed during culture maintenance, so the calculated injection volume was determined daily prior to administering the food supply. We injected food algae between 3 and 4 pm.

**Table 1:** Size and density of two food algal species used in the experiment.

Prey species	Size (µm)	Density (cells mL^−1^)
***C. vulgaris***	3.84±0.6	4333.3±629.1
***S. hantzschii***	4.03±0.4	687.3±142.3

Density of the two algal species was the average daily dose provided to *Daphnia magna* in the growth experiment.

Unlike supplying a single algal species, for one treatment (MIX), a mixture of two food algae was used. In the MIX group, careful determination of the food algal supply was performed. To maintain the daily dosage of food algae at 2.5 mg C L^−1^ for the two different algal species, each species was supplied at 1.25 mg C L^−1^. We determined the required quantity of each algal species by daily enumeration of food algal density.

The experiment was conducted using a plant growth chamber (Eyela FLI-2000, Japan). The aforementioned maintenance conditions for *D. magna* were also applied to the experiment (20°C; photon flux density = 30 µmol·m^−2^·sec^−1^; 12L:12D light-dark cycle). The experiment was terminated when adult *D. magna* produced offspring from the second clutch. The shortest duration in which the second clutch was obtained was 8 d, which is generally accepted as an appropriate turnover time for the assimilation of carbon and nitrogen from *D. magna* food [26]. Each day during the experiment, we transferred *D. magna* to fresh Elendt M4 medium before providing food algae, to maintain the supply of algae at 2.5 mg C L^−1^. After termination of the experiment, we randomly collected two adults from each beaker of the three experimental groups (total *n* = 20 for each experimental group), and measured their body length and counted food algal cells in their guts. We took microscope images (×200 magnification, Axioskop 40, Carl Zeiss Microscopy, Germany) of the adult *D. magna* and used an image processing program (AxioVision Rel 4.8, Carl Zeiss Microscopy, Germany) to measure body length following the manufacturer’s protocol for calibrating image length to actual length.

Gut contents were examined to determine the pattern of food algal consumption, particularly in the MIX group. For this investigation, we eviscerated the guts of *D. magna* (*n* = 20) from the MIX group and counted algal cell numbers of each species in the guts of each individual. Food algal cells tended to be broken as they progressed through the gut, which could complicate enumeration of algal cells. From an empirical approach, just after consumption, algal cells resided in the upper part of the gut (approximately 1 mm from the mouth) and were relatively fresh and unbroken. Therefore, we divided the total gut length (approximately 3 mm) into three sections (fore-, mid-, and rear-gut; each approximately 1 mm), and counted algal cells in the foreguts to minimize enumeration error due to broken cells. Typically, filter feeders such as daphniids are not selective feeders; therefore, the ratio of the two consumed algal species in the foregut would be maintained during passage through the gut.

To determine offspring size, two randomly sampled offspring from each beaker (total *n* = 20 in each experimental group) were measured. The size measurement of offspring was based on application of the image-processing program, as performed for the size analysis of *D. magna* adults. To determine the total number of offspring per adult, we counted the number of offspring in each beaker and divided that number by five (i.e., the number of adults in each beaker).

The density and size measurements of zooplankton individuals and algal cells in the gut samples were carried out using a microscope (Axioskop 40, Carl Zeiss Microscopy, Germany) under ×200, and ×400 magnification, respectively.

### Stable Isotope Analysis

The remaining 30 adult *D. magna* individuals in each experimental group and the two food algae were used for stable isotope analysis. The *D. magna* samples contained phytoplankton in their guts; therefore, we transferred those individuals into fresh Elendt M4 medium for more than 24 h without provision of additional food algae. This allowed these individuals to eject their gut contents; they were then included in the stable isotope analysis. The 30 *D. magna* individuals were divided into six groups (*n* = 5 per group); three of these groups (*n* = 15 individuals) were used for detection of the carbon signature and three groups were used for detection of the nitrogen signature.

Carbon and nitrogen measurements of *D. magna* were conducted separately. It is necessary to extract tissue lipids for accurate interpretation of trophodynamics using carbon stable isotope data. The carbon isotope signature depends on protein content in tissue; the presence of lipids can affect the reliability of the isotope analysis. Lipid content varies in accordance with tissue type and is ^13^C-depleted relative to proteins. Therefore, tissue samples that contain lipid may produce an unstable carbon isotope signature. In contrast, lipid extraction affects δ^15^N. Therefore, we divided the samples into separate groups comprising carbon- and nitrogen-signature samples. Lipids were removed only from the carbon-signature samples. Comparison between the two samples was accomplished by δ^13^C and δ^15^N analyses [27]. The carbon-signature samples were placed in a solution of methanol-chloroform-triple-distilled water (2∶1:0.8 v/v/v) for 24 h.

For stable isotope analysis of food algae, we prepared 5 mL of algal suspension from each species and analyzed in triplicate. The algal samples were treated with 1 mol·L^−1^ hydrochloric acid (HCl) to remove inorganic carbon. The samples were then rinsed with ultrapure water to remove the HCl.

The prepared samples (two algal species and *D. magna*) were freeze-dried and then ground with a mortar and pestle. The powdered samples were maintained at–70°C until analysis. When all samples were collected, carbon and nitrogen isotope ratios were determined using continuous-flow isotope mass spectrometry. Dried samples (approximately 0.5 mg of animal samples and 1.0 mg of algae) were combusted in an elemental analyzer (EuroVector), and the resultant gases (CO_2_ and N_2_) were introduced into an isotope ratio mass spectrometer (CF-IRMS, model-ISOPRIME 100, Micromass Isoprime) in a continuous flow, using helium as the carrier gas. Data were expressed as the relative per-mil (‰) difference between sample and conventional standards of Pee Dee belemnite (PDB) carbonate for carbon and air N_2_ for nitrogen, according to the following equation:
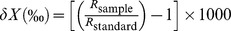
where X is ^13^C or ^15^N, and R is the ^13^C:^12^C or ^15^N:^14^N ratio. A secondary standard of known relation to the international standard was used as a reference material. The standard deviations of δ ^13^C and δ ^15^N for 20 replicate analyses of the ‘Peptone (δ^13^C = −15.8‰ and δ^15^N = 7.0‰, Merck)’ standard were ±0.1 and ±0.2 (‰), respectively.

To determine which of the two food sources (*C. vulgaris*, and *S. hantzschii*) was assimilated more readily by *D. magna*, we calculated two-source isotope mixing models. The carbon isotope values of *C. vulgaris* and *S. hantzschii* differed significantly. The model is defined as:
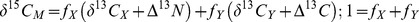
where X, Y, and M represent the two food sources and the mixture, respectively; *f* represents the proportion of N from each food source in the consumer’s diet; and Δ^15^C is the assumed trophic fractionation (i.e., the change in δ^ 15^C over one trophic step from prey to predator) [28], [17]. Trophic fractionation was assumed to be constant, and either 3.4‰ or 2.4‰ [29].

### Statistical Analysis

For statistical assessment of the experimental groups, we applied one-way nested ANOVA (two-tailed, α = 0.05) to compare the size of adults and offspring. Although we prepared 10 replicates (beakers) for each experimental group, pseudo-replication had to be carefully considered [30]. Therefore, we set the different food algal treatments as the primary factors and the 10 beakers as nested subgroups for every treatment.

Comparison of numbers of *D. magna* offspring was performed using one-way ANOVA. Student’s *t*-tests (two-tailed, α = 0.05) were used to compare cell numbers of algal species in the guts of the MIX group. Tukey’s post-hoc tests were employed to identify groups with different average values. All statistical tests were performed using the package SPSS Statistics ver. 20.

## Results

### *D. magna* Response to Different Food Algae Resources

**Figure 2:**
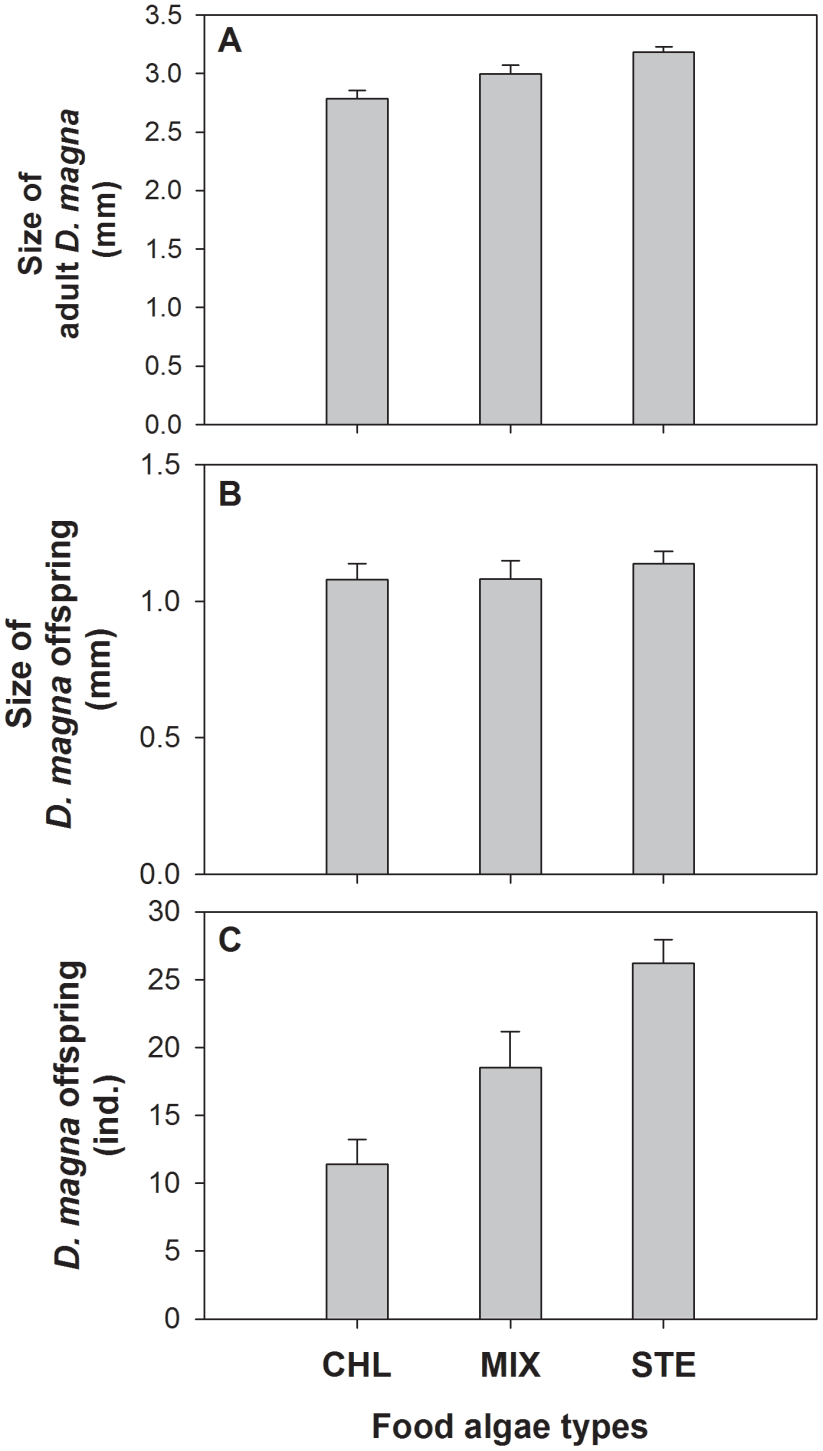
Size and number of individual *Daphnia magna* according to the prey species provided. A) size of adults; B) size of offspring; C) number of individual offspring. CHL, *C. vulgaris*; STE, *S. hantzschii*; MIX, a mixture of both food algal species; *n* = 20, respectively.

A clear difference in size of adult *D. magna* was observed between the experimental groups (Figure 2 and Table 2). Adult *D. magna* that consumed *S. hantzschii* were significantly larger than those that fed on *C. vulgaris* (mean ± standard deviation; STE: 3.18±0.05 mm; CHL: 2.78±0.07 mm). Adult size in the MIX group was intermediate (2.99±0.07 mm; Figure 2A). Although subgroups (i.e., beakers) showed statistical differences, post-hoc tests revealed a significant difference in average values of the three groups (three comparisons, *P*<0.001). The size of *D. magna* offspring also differed significantly between groups (Figure 2B and Table 2). As for adults, offspring from the STE group (1.14±0.05 mm) were significantly larger than offspring from the other groups (CHL: 1.08±0.06 mm; MIX: 1.08±0.07 mm). The sizes of individuals in the CHL and MIX groups were similar. Post-hoc tests revealed significant differences between CHL and STE, and between MIX and STE (*P*<0.001), but not between CHL and MIX (*P*>0.05).

**Table 2:** Two-way nested ANOVA results for the effects of main groups (i.e. food algae *Chlorella vulgaris*, *Stephanodiscus hantzschii,* and Mixture) and subgroups (i.e. beakers) on adults and offspring of *D. magna* size.

	Factors	d.f.	F	*p*
**Size of adult ** ***D. magna***	Food algae	2	91.85	P<0.001
	Beaker	18	2.95	P<0.01
**Size of ** ***D. magna*** ** offspring**	Food algae	2	5.67	P<0.05
	Beaker	18	0.91	P>0.05

Difference in food algae also resulted in variations in offspring number (Figure 2C). Adult *D. magna* that consumed *S. hantzschii* produced more offspring than did those of the other groups (STE: 26.2±1.8 ind. per adult; CHL: 11.4±1.8 ind. per adult; MIX: 18.5±2.67 ind. per adult). The three groups differed significantly from one another (one-way ANOVA; F = 120.76, *P*<0.001, d.f. = 2), which was supported by the results of post-hoc tests (all three cases, *P*<0.001). Gut content analysis showed that *D. magna* adults in the MIX group consumed both *C. vulgaris* and *S. hantzschii* during the experiment (*C. vulgaris*, 1,966.0±235.3 cells per gut; *S. hantzschii*, 2,130.0±460.4 cells per gut; t = –1.457, *P*>0.05; *n* = 20).

### Stable Isotope Analysis of Food Algae Assimilation

**Figure 3:**
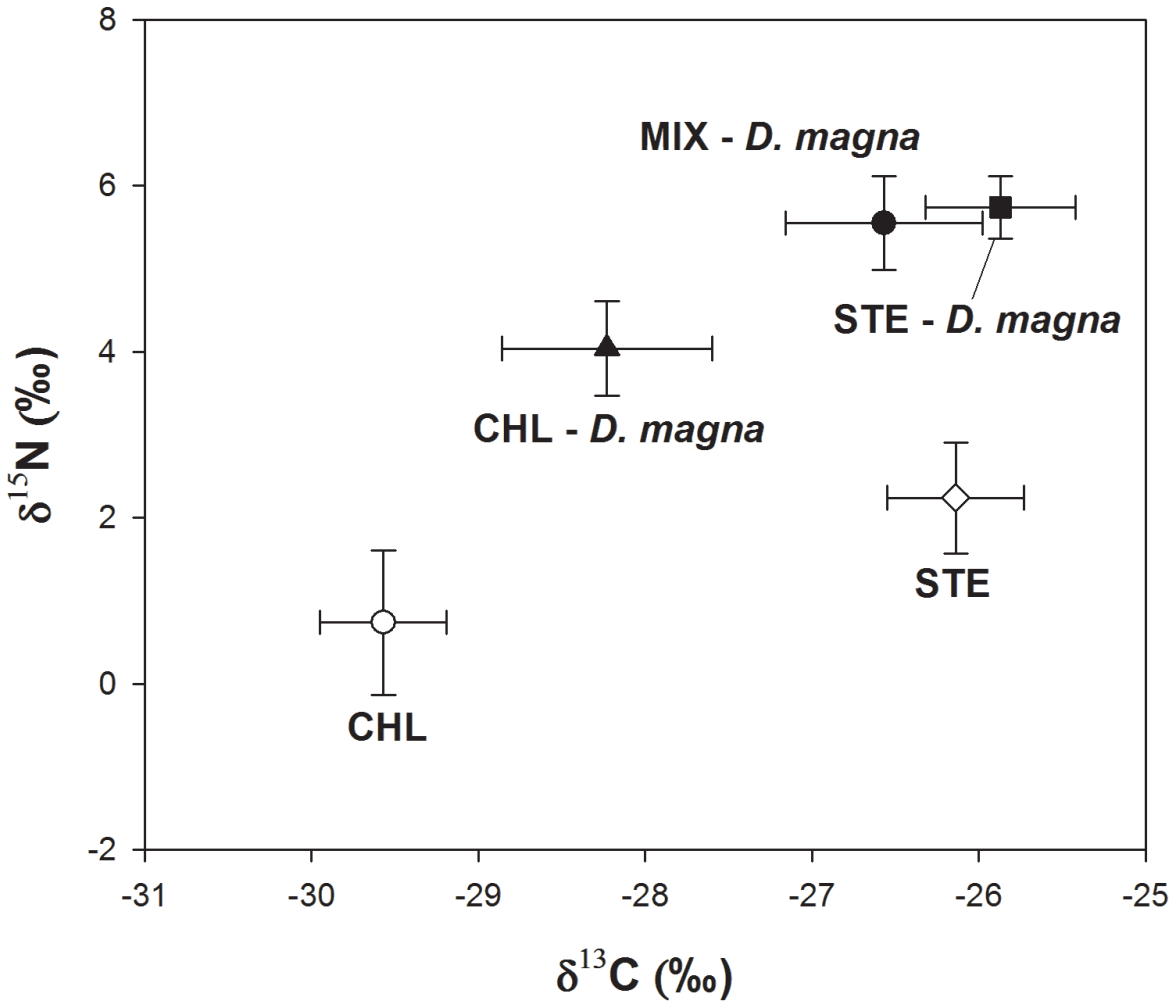
Results of the stable isotope analysis. CHL, *C. vulgaris*; STE, *S. hantzschii*; MIX, a mixture of both prey species.

Stable isotope analysis revealed that *D. magna* depended more on *S. hantzschii* than on *C. vulgaris* when they fed on a mixture of these algae (Figure 3). The δ^13^C and δ^15^N ratio indicated the contribution of prey phytoplankton to *D. magna*. *D. magna* adults in the two groups fed only one species (CHL and STE) depended on either *C. vulgaris* or *S. hantzschii*, respectively. However, *D. magna* in the MIX group relied more on *S. hantzschii*. In addition, when the contribution rates of the two food algal species in the MIX group were calculated from the isotope analyses, the *S. hantzschii* contribution rate (92%) was higher than that of *C. vulgaris* (8%) from the two-source mixing model. Therefore, diatom species (*S. hantzschii*) contributed most to the growth of *D. magna* individuals and populations.

## Discussion

### Contribution of Different Algal Species to *D. magna* Growth

Of the two algal species studied, the diatom *S. hantzschii* appears to be the more suitable food item for *D. magna*; this was true for both population growth and for individual growth. Despite similar consumption rates of the two algae (see Table 1), the size of *D. magna* individuals increased much more when they utilized *S. hantzschii*. There are several explanations for why this may occur. Diatoms are commonly regarded as good sources of lipids and serve as a food source for zooplankton [31], [8]. They are known to contain large amounts of eicosapentaenoic acid [32], a fatty acid required in the diet of many animals that may not be able to synthesize the compound [33], [34]. However, green algae are known to contain relatively lower nutrient contents compared to diatoms.

However, a greater availability of food algae (quantity-wise) does not always guarantee increased growth of grazer populations. A second possibility involves the digestive capacity of *D. magna*. The digestion rate of *D. magna* differs depending on the phytoplankton species consumed. Van Donk et al. [35] suggested that the cell wall morphology of green algae might reduce their digestibility by *Daphnia*. Our stable isotope results suggest that the greater dependence of *D. magna* on *S. hantzschii* is attributable to more effective absorption of nutrients from the diatom species. Therefore, it may be assumed that a balance between quality and absorbability of food algae is important for individual and population growth in zooplankton. Further research should investigate the characteristics of this balance.

### Offspring Characteristics

An interesting finding of this experiment was the changing pattern of offspring size and number according to the species of algae consumed. Both abundance and size of offspring in the STE group were greater than in the other groups. Although the size and number of offspring depend on clutch size and reproductive capability of adults [36], we suggest that *D. magna* adults may respond flexibly to the quality of the energy sources they capture, resulting in changes in the size and number of their offspring in accordance with different algal species consumed. That *D. magna* actively responds to food algal quality was not definitively shown, but the response was clear. When algal resources are of low quality (less nutritious algae, such as *C. vulgaris* in the present study), female *Daphnia* may limit offspring size to ensure survival. Enhanced nutritional conditions (inclusion of *S. hantzschii* in the MIX group) resulted in a slight increase in offspring number. We maintained the supported amount of carbon in all of treatments at 2.5 mg C L^−1^, but the algal species that comprised this carbon differed. Although availability of carbon allowed survival and reproduction of *D. magna*, offspring characteristics were further improved when the proportion of *S. hantzschii* was increased. The provision of more nutritious food algae caused this pattern to emerge. We thought that a semi-restricted diet would result in moderate changes in size and fecundity (i.e., the MIX group) and that a nutritious diet fed to a growing individuals would increase the size and fecundity of that individual, thereby also increasing the population (i.e., the STE group). In previous studies, offspring size was affected by the quantity of food algae and the presence of predators [37], [38]. Although we did not consider the presence of predators, the quality of food algae plays a key role in the population growth of *D. magna*, at least when food algae are sufficiently abundant.

The quality of food algae may be very important to filter-feeding zooplankton. Recent studies have found that other zooplankton groups (mainly copepods) are not affected by the quality of algae resources during population growth [39]. In one study, egg production was not significantly related to lipid content in six phytoplankton species; the authors suggested that slow transit time in the gut (i.e., increased opportunity to absorb nutrients) could explain this result. In contrast to copepods, *Daphnia* typically shows relatively fast gut-passage time, which does not allow optimal absorption of nutrients [40]. Therefore, the quality of food algae, as well as its absorbability, is crucial for *Daphnia* population growth, and food algae that are fully assimilated will result in maintenance of or increases in zooplankton population levels.

### Appropriate Food Selection using Stable Isotope Analysis

Based on the results of this study, it is possible to quantify the energy channeled from primary producers to primary consumers, expanding on basic understandings of connectivity. The traditional method of investigating food web structure involves visual inspection of gut contents [41], [42], but recently, DNA barcoding has increased the resolution of prey identification to the species level [43]. From a functional perspective, prey consumption is related to the growth of grazers and predators [44], [45]. Despite apparent evidence, these methods are limited in their ability to quantify the contribution of prey to grazers. Assimilation indicates how grazers utilize prey for growth, and the results of the present study further elucidate microbial food web structure. Consequently, the consumption rate (including qualitative and quantitative aspects) and the contribution rate of food items should be examined simultaneously to further precisely elucidate food web functions. In addition, as more information on multi-species prey and grazer relationships becomes available, understanding of ecological integrity will be improved.

## Conclusion

The growth of *D. magna* individuals and offspring was significantly improved by the consumption of *S. hantzschii* but not *C. vulgaris*. Stable isotope analysis revealed substantial assimilation of diatom-derived materials in *D. magna*, indicating that diatoms are important to the population growth of this species. These results confirm the applicability of stable isotope approaches for clarifying the contribution of different food algae and for elucidating the importance of food quality for *D. magna* population growth.
